# The Contribution of Macrophage Plasticity to Inflammatory Arthritis and Their Potential as Therapeutic Targets

**DOI:** 10.3390/cells13181586

**Published:** 2024-09-20

**Authors:** Karina Kulakova, Tope Remilekun Lawal, Eoghan Mccarthy, Achilleas Floudas

**Affiliations:** 1 School of Biotechnology, Dublin City University, D09 V209 Dublin, Ireland; karina.kulakova4@mail.dcu.ie (K.K.);; 2 Life Sciences Institute, Dublin City University, D09 V209 Dublin, Ireland; 3 Department of Rheumatology, Beaumont Hospital, D09 V2N0 Dublin, Ireland; 4 Royal College of Surgeons in Ireland, D02 YN77 Dublin, Ireland; 5 Medical School, University of Ioannina, 45110 Ioannina, Greece

**Keywords:** inflammatory arthritis, rheumatoid arthritis, macrophage polarisation, plasticity

## Abstract

Inflammatory arthritis are common chronic inflammatory autoimmune diseases characterised by progressive, destructive inflammation of the joints leading to a loss of function and significant comorbidities; importantly, there are no cures and only 20% of patients achieve drug-free remission for over 2 years. Macrophages play a vital role in maintaining homeostasis, however, under the wrong environmental cues, become drivers of chronic synovial inflammation. Based on the current “dogma”, M1 macrophages secrete pro-inflammatory cytokines and chemokines, promoting tissue degradation and joint and bone erosion which over time lead to accelerated disease progression. On the other hand, M2 macrophages secrete anti-inflammatory mediators associated with wound healing, tissue remodelling and the resolution of inflammation. Currently, four subtypes of M2 macrophages have been identified, namely M2a, M2b, M2c and M2d. However, more subtypes may exist due to macrophage plasticity and the ability for repolarisation. Macrophages are highly plastic, and polarisation exists as a continuum with diverse intermediate phenotypes. This plasticity is achieved by a highly amenable epigenome in response to environmental stimuli and shifts in metabolism. Initiating treatment during the early stages of disease is important for improved prognosis and patient outcomes. Currently, no treatment targeting macrophages specifically is available. Such therapeutics are being investigated in ongoing clinical trials. The repolarisation of pro-inflammatory macrophages towards the anti-inflammatory phenotype has been proposed as an effective approach in targeting the M1/M2 imbalance, and in turn is a potential therapeutic strategy for IA diseases. Therefore, elucidating the mechanisms that govern macrophage plasticity is fundamental for the success of novel macrophage targeting therapeutics.

## 1. Introduction

Inflammatory arthritis (IA) is a diverse group of chronic inflammatory autoimmune diseases that includes Rheumatoid Arthritis (RA), Psoriatic Arthritis (PsA), Ankylosing Spondylitis and Juvenile Idiopathic Arthritis (JIA) and these are characterised by common inflammatory-related symptoms such as redness, swelling, warmth, pain and joint dysfunction [1,2]. Chronic IA is histologically characterised by a hyperplasia of the intimal lining layer of the synovial membrane and infiltration of the synovial sublining [3]. IA affects over 1% of the global population with an incidence rate ranging from 115 to 271 per 100,000 adults [4,5]. In most cases of IA, the disease is systemic. If left untreated, IA can lead to significant joint deformation and damage. IA arises from an abnormal immune response targeting the synovial membrane lining joints, in response to harmful stimuli, such as pathogens, damaged cells and toxic compounds, which induce acute and/or chronic inflammatory responses [6]. The precise aetiology of IA is uncertain, however, genetic predisposition, environmental factors and dysregulated immune responses contribute to its development [7,8].

### 1.1. Inflammatory Arthritis Pathogenesis

RA involves multiple joints of the body and is characterised by tenosynovitis due to tendon inflammation resulting in bone erosion and cartilage destruction [9]. During inflammatory joint disease, an increased number of mononuclear phagocytes and synovial fibroblasts (SFs) contribute to an expanding synovial pannus, unusual tissue formation resulting from heightened inflammation triggered by elevated levels of cytokine and immune cells, which drives the destruction of articular cartilage and bone [10,11]. Pannus formation is a hallmark of RA leading to increased receptor activator for nuclear factor κ-B ligand (RANK-L) expression, osteoclast (OC) activation and bone erosion [10]. A progressive symmetric inflammation of the affected joints can be observed. RA-affected joints typically lack signs of repair, which in turn contributes to a rapid loss of joint structure [12]. The systemic and organ-specific complications of the disease are associated with a high incidence of morbidities and rising mortality [13].

RA is a heterogeneous disease displaying variability in clinical presentation and pathogenetic mechanisms across disease stages and in individuals of the same formal diagnosis [14]. The disease is characterised by rheumatoid factor (RF); IgM autoantibodies against IgG and anti-citrullinated protein antibody (ACPA) [8]. Individuals displaying autoantibodies are described as having seropositive RA and those negative for RF and ACPA are described as having seronegative RA. Patients positive for anti-carbamylated protein (Anti-CarP) autoantibodies are associated with a more active disease [15]. Carbamylation is a non-enzymatic process which generates homocitrulline when cyanate ions react with amino groups of arginine and lysine [16]. The combination of anti-CarP and ACPA positivity observes a higher risk of developing bone and cartilage erosion [15]. Autoantibody-positive IA patients display a worse prognosis, more radiographic damage and a lower chance of achieving drug-free remission [17].

RA has a strong genetic component with heritability at approximately 60% in ACPA-positive patients [8]. A family history of RA increases the risk of developing the disease three to five times [8]. In addition to this, concordance risk rates in identical twins are 15% higher than in non-identical twins or non-related individuals [18]. Genome-wide association studies have found over 100 loci of single nucleotide polymorphisms associated with RA of which many are shared with other IA diseases [19]. The strongest gene association with RA is considered to be in class II human leukocyte antigen (HLA), in particular the HLA-DRB1 alleles (DRB1*01 and DRB1*04) which comprise approximately 40% of the observed genetic susceptibility [8]. These alleles form an important gene–environment interaction in which the presence of the HLA-DRB1 alleles combined with smoking form a significant risk factor for ACPA-positive RA [8]. Common environmental factors triggering RA inflammatory conditions include smoking, obesity, *Porphyromonas gingivialis* (*P. gingivialis*) infection, synovial injury and the hyperplasia of SFs. Periodontitis infection manifested by the common periodontal bacterium *P. gingivialis* is strongly associated with RA development. The bacteria induce autoimmune responses by the citrullination of host peptides catalysed by protein arginine deiminase (PAD) enzymes [20]. This process results in a net loss of charge through the conversion of positively charged arginine residues of “self” proteins into neutral citrulline residues. This breach of local tolerance by *P. gingivalis* promotes the susceptibility of neoepitope generation, protein degradation and the downstream generation of ACPAs [20,21]. The strongest association between RA and smoking is ACPA-positive disease in individuals with at least one copy of the shared HLA-DRB1 epitope [7,8]. The interaction between smoking and the epitope can increase risk by 20-fold compared to non-smokers without the epitope [7]. Exposure to the toxic components of cigarette smoke results in the depression of antibacterial and phagocytic functions of alveolar macrophages and their inability to kill intracellular bacteria [7]. This results in a local breach of tolerance as alveolar macrophages are crucial to the homeostasis of the inflammatory environment in the lung [22]. Hence, the environmental factors involved in RA trigger the development and establishment of disease in genetically susceptible individuals.

PsA is a chronic IA disease characterised by chronic synovial inflammation and joint destruction, which typically occurs in patients with psoriasis [23]. The diseases display characteristics of progressive and destructive changes. Patients with PsA manifest joint inflammation, enthesitis, dactylitis, skin manifestations, cardiovascular diseases and other comorbidities [24]. The prevalence of PsA among psoriasis patients varies between 6% and 42% [25]. The disease holds complex and multifaceted pathogenesis with genetic predisposition and environmental factors, such as smoking, bacterial infection and obesity, triggering an inflammatory response and releasing pro-inflammatory cytokines [23]. In PsA, inflammation involves increased blood vessel formation and immune cell infiltration which damage cartilage and bone by invading the synovial tissue and releasing enzymes which degrade the extracellular matrix (ECM) [26]. A key difference between RA and PsA is in their patterns of joint pathology as highlighted in Table 1 [12]. RA is characterised by progressive joint and bone destruction, whereas PsA is characterised by new bone formation [12]. After the initial destructive process of PsA, affected joints attempt to compensate for the damage by forming osteophytes [12]. These are bony projections originating from the periosteum membrane lining. Osteophyte formation ultimately leads to the stiffening and immobilisation of the inflamed joint [12]. Interleukin (IL)-17 involvement and expression levels are similar for both RA and PsA patients [27]. This cytokine is a transcriptional activator of pro-inflammatory cytokines, acute-phase response genes, hematopoietic cytokines and antimicrobial mediators which work in synergy with IL-1β and tumour necrosis factor (TNF) [27]. Cytokines released by immune cells activate fibroblast-like synoviocytes (FLSs) which leads to the differentiation of monocyte progenitor cells into OCs, contributing to bone tissue breakdown which typically occurs away from the cartilage–pannus junction [28]. This self-sustaining process eventually results in joint deformations and a loss of function [28]. Recent single-cell RNA sequencing (scRNA-seq) analysis of synovial tissue biopsies from RA and PsA patients uncovered a synergic interaction between synovial macrophages and T cells, facilitated by IL-1β and TGF-β, which influence the gene expression profile of pro-inflammatory SFs specifically in RA, not in PsA [29]. The onset of PsA shows diverse tissue origin, presenting heterogeneous symptoms and variable disease courses and outcomes [23]. Understanding these distinctive features is crucial in comprehending the complexity of these arthritic conditions and devising effective pathogenesis and treatment strategies.

### 1.2. Currently Available Therapeutics

While novel biotherapeutics have improved IA management, there are no cures. It is difficult to predict the treatment outcome for patients, such as who will develop severe, erosive diseases or who will respond to treatment. Clinical and genetic differences in disease activity or duration are not reliable predictors of treatment response or therapeutic targets [5]. As a result, patients are undergoing several rounds of treatment regimens until the right treatment is identified. This results in severe debilitating trauma for patients and comorbidities associated with the treatments. Hence, the use of targeted biomarker screening is vital for the identification of suitable treatments and further research is required in IA for the development of new targeted therapeutics [38].

## 2. The Role of Macrophages in Chronic Inflammation

Bone and joint remodelling are characteristic of IA and are regulated by the Wnt pathway, specifically the Dickkopt-1 (DKK-1) negative regulator [12]. DKK-1 reduces the Wnt signal when bound to the LPR5/6 receptor and Kremen-1/2 [12]. DKK-1 expression in IA inhibits bone formation in the joint, thereby further favouring joint instability [12].

IA diseases involve components of both the innate and adaptive immune system with dysregulated cytokine production significantly contributing to disease onset and progression [3]. The synovium is the primary site of inflammation associated with RA [2]. Macrophages play an important role in the innate immune system’s inflammatory response by contributing to the development of intimal lining layer hyperplasia, a condition characterised by the thickening of the innermost blood vessel layer [3,39]. They are present on several body cavities, tissues and around mucosal surfaces. Macrophages are pluripotent cells that function as antigen-presenting cells with important roles in pathogen clearance, inflammation resolution and wound healing [40]. Macrophage staining, specifically, the histological evaluation of CD68 in synovial tissue biopsies, has shown a correlation with disease severity [41]. Angiogenesis occurs with persistent synovitis causing increased inflammatory macrophage recruitment to the synovium where they secrete more inflammatory mediators to exacerbate synovitis [2]. Synovitis is morphologically characterised by synovial hyperplasia which is an increased accumulation of macrophages in the intimal lining of the synovium and positively correlates with RA activity [2]. Macrophages are a source of pro-inflammatory cytokines and chemokines, activate immune cells and secrete various tissue-degrading enzymes, leading to chronic pro-inflammatory, pain and tissue-destructive responses in RA [39]. However, they can also function to produce anti-inflammatory cytokines and promote immunomodulatory and protective responses.

**Figure 1:**
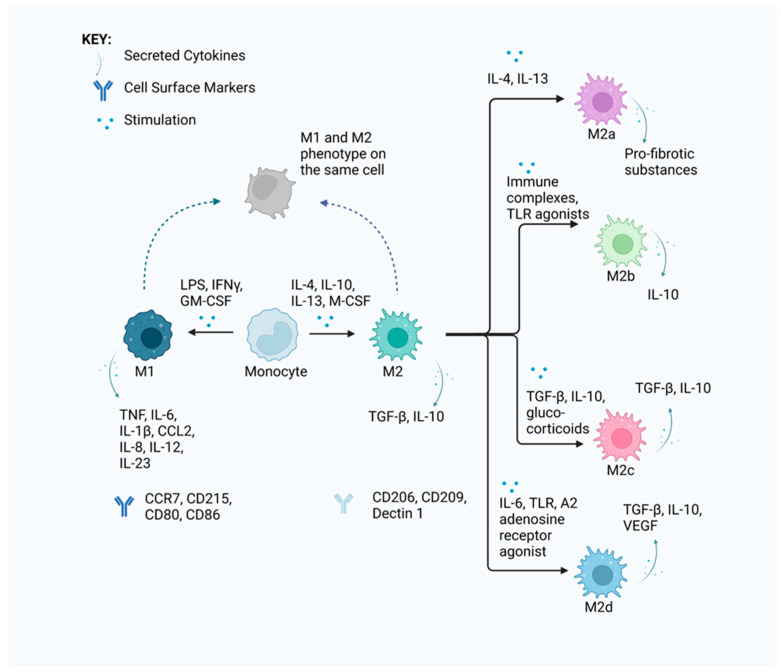
Macrophage polarisation conditions. This schematic illustrates macrophage polarisation, depicting distinct functional phenotypes (M1, M2a, M2b, M2c and M2d) in response to microenvironmental signals. Each polarisation state is characterised by specific markers and cytokines contributing to either pro-inflammatory or anti-inflammatory responses.

Macrophages can originate from adult circulating monocytes or the yolk sac during embryonic development [42]. Their polarisation can be influenced by various factors, including the nature of the stimulus, transcription factors (TFs), epigenetics, the tissue microenvironment and the presence of other immune cells, and thereby shape their subsequent immunological response to activating stimuli [3]. Key TFs such as PU.1, C/EBPα and IRF8 play roles in macrophage development and function by activating specific gene expression and maintaining macrophage-specific enhancer elements [43]. PU.1 and IRF8 are involved in the monocytic lineage-specific expression of the Nod-like receptor pyrin domain containing 3 (NLRP3) pattern recognition receptor (PRR) [43]. The chronic activation of the NLRP3 inflammasome is involved in the onset and progression of IA [43]. This is achieved by the inflammasome complexing with pro-Caspase-1, converting it into Caspase-1 which in turn cleaves pro-IL-1β into its active form [43]. As a result, the TFs promote an inflammatory environment in the body and their knockdown can be utilised for the downregulation of NLRP3 [43]. Remarkable diversity is exhibited by macrophages in their origin, function and metabolism, with environmental factors playing a role in their specialisation [44]. Their polarisation is a dynamic process in which macrophages can adopt different functional phenotypes ranging from pro-inflammatory (M1) to anti-inflammatory (M2) in response to signals from their microenvironment, including chemokines, cytokines and FLSs, as summarised in Figure 1 [29,44]. Different metabolic processes are induced between M1 and M2 macrophages, which play significant roles in their respective phenotypes [44].

**Figure 2:**
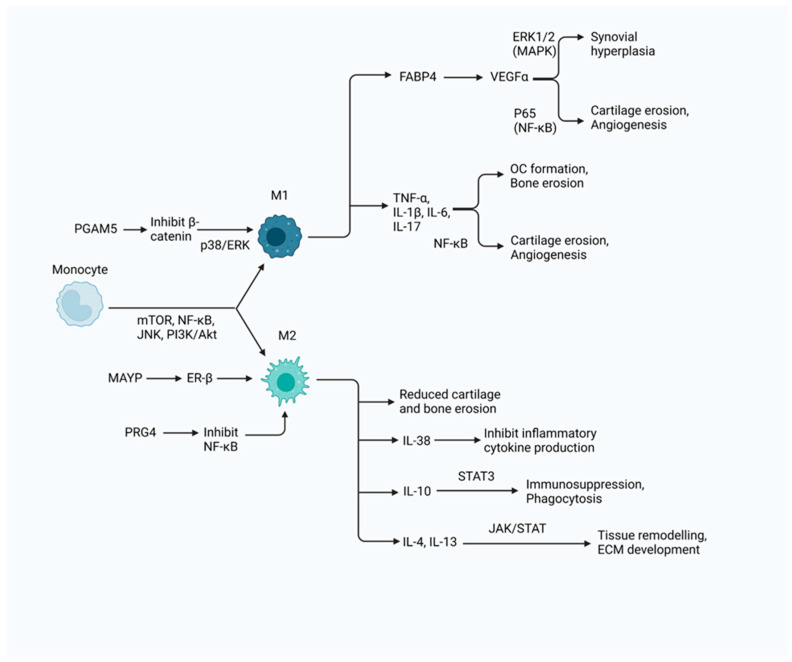
Potential pathways of macrophage contribution to synovial inflammation. M1 macrophages exert pro-inflammatory activity in the synovium via the NF-κB and MAPK signalling pathways and secretion of pro-inflammatory cytokines and chemokines. This leads to OC formation, angiogenesis, synovial hyperplasia, bone and cartilage erosion and degeneration. M2 macrophages produce anti-inflammatory effects in the synovium via anti-inflammatory cytokine expression and the JAK/STAT pathway. As a result, M2 macrophages are involved in wound healing, tissue remodelling and inflammation resolution and lead to homeostasis in the synovium.

Most scRNA-seq studies regarding macrophages in RA patients have aimed to understand the polarisation states and molecular signatures of these cells in the synovium of RA patients. In one such study, Li et al. utilised scRNA-seq, flow cytometry and immunofluorescence to investigate the expression of CD86 and CD206 in macrophages [45]. The upregulation of both markers was observed in macrophages of the synovium, in particular the lining layer [45]. Three clusters of macrophages were detected: nonpolarized, inflammatory M1 and anti-inflammatory M2 macrophages. Non-polarised macrophages exist at the start of the differentiation trajectory [45]. Under RA conditions, all three subpopulations upregulated CXCL1, CXCL2, CXCL3, CCL4, CCL4L2, IL-1β, TNFAIP3, ICAM1, PLAU and TNF in the NF-κB signalling pathway to varying extents [45]. Li et al. found that nonpolarized macrophages expressed NUPR1 and C1QA, M1 macrophages expressed IL-1β and SPP1, and M2 macrophages expressed MerTK [45]. Lining layer macrophages were characterised by high TREM2 expression [11,45]. Depending on the macrophage phenotype, they contribute to synovial inflammation through various pathways, as seen in Figure 2, and exert pro- or anti-inflammatory activity in the synovium.

### 2.1. M1 Macrophages

#### 2.1.1. Phenotype

Classically activated macrophages (M1) possess a pro-inflammatory phenotype [46]. These macrophages are known for causing significant joint erosion through their secretion of pro-inflammatory cytokines such as TNF, IL-6, IL-1β, IL-12, IL-23 and C-C motif chemokine ligand 2 (CCL2) and reactive oxygen species (ROS) [9,32]. The M1 macrophage phenotype is further characterised by a high production of reactive oxygen and nitrogen intermediates, strong tumoricidal and microbicidal activity and the promotion of Th1 responses [47]. M1 macrophages are characterised by the expression of phenotypic markers, such as Toll-like receptors (TLR2 and TLR4) and CCR7, CD215, CD80 and CD86 [32,33]. They are involved in triggering inflammation and tissue damage [48]. The activation of the TLR-4-induced nuclear factor kappa-B (NF-κB) signalling pathway mediates the pro-inflammatory activity of M1 macrophages via TNF-α, IL-1β and IL-6 production and release in monocyte-derived (MDMs) and synovial macrophages in patients with RA [46]. In addition to this, nitric oxide (NO) formation is a fundamental feature of M1 macrophages followed by the upregulation of inducible nitric oxide synthase 2 (NOS2) [9,46,49,50]. An enhanced expression of NOS2 has been observed in the synovium of patients with IA [51]. NO formation leads to the suppression and loss of mitochondrial electron transport chain (ETC) complexes, which is implicated in M1 macrophage decreased metabolic mitochondrial activity [49].

#### 2.1.2. Polarisation Conditions

Macrophage polarisation in the inflamed joint is not well understood; however, there is an evident imbalance of cytokine activity favouring the pro-inflammatory M1 macrophages [46,51]. Macrophage polarisation takes place in response to an immunological stimulus or in order to repair damaged tissue [40]. The polarisation of macrophages towards an M1 or M2 phenotype is a precisely regulated process consisting of several key signalling pathways, transcriptional epigenetic and post-transcriptional regulatory networks [52]. Monocytes differentiate into M1 macrophages in the presence of granulocyte-macrophage colony-stimulating factor (GM-CSF) [53]. Classical inflammatory monocytes have TLRs and scavenger receptors on their surface which aid in the identification of pathogen-associated molecular patterns (PAMPs) and the production of effector molecules such as cytokines to initiate inflammation [54]. They are selectively trafficked to the site of inflammation where they contribute to local and systemic inflammatory responses [55]. These monocytes are highly infiltrative and can differentiate into inflammatory M1 macrophages which remove PAMPs and cell debris [55]. TFs such as NF-κB, signal transducer and transcription activator 1 (STAT1) and IRF-5 are involved in M1 polarisation [9]. They further polarise and become activated by interferon-γ (IFNγ) and lipopolysaccharides (LPSs) [32]. IFNγ is critical for macrophages to polarise into the M1 phenotype as it triggers specific gene expression profiles, such as the major histocompatibility complex (MHC) II, and activates Janus kinase (Jak) adapters leading to subsequent STAT1 activation [56,57]. PRRs activate TLR4 which in turn activates the myeloid differentiation response 88 (MyD88) and NF-κB pathway [58]. In macrophages, the conditional depletion of microRNAs (miRs)-processing enzyme Dicer induces M1-like tumour-associated macrophage (TAM) expression, marked by intensified IFNγ/STAT1 signalling [52]. In combination, these processes lead to the expression of cytokines which lead to an inflammatory environment.

Phosphoglycerate mutase 5 (PGAM5) has been implicated in enhancing M1 polarisation in macrophages through the Akt-mTOR/fls/ERK pathways [51]. PGAM5, found in the mitochondrial membrane, is a critical regulator of mitochondrial metabolism and dynamics, modulates cellular senescence and enhances inflammasome activation in macrophages [51]. PGAM5 inhibits M2 macrophage polarisation via the STAT6-PPARγ pathway [51]. A conditional knockout of PGAM5 results in the alleviation of IA symptoms through the repolarisation of M1 macrophages into M2 macrophages [51]. PGAM5 can regulate macrophage polarisation by inhibiting β-catenin via the dephosphorylation of dishevelled segment polarity protein 2 (DVL2) [51]. This occurs through the direct binding of PGAM5 to DVL2. The inhibition of the β-catenin pathway enhances the M1 phenotype through the activation of the p38/ERK signalling pathway and inhibition of the STAT6-PPARγ pathway of the M2 response [51]. The stimulation of macrophages with LPS and IFNγ, leading to their polarisation into the M1 phenotype, demonstrates a significantly activated Akt-mTOR signalling pathway [51]. The activation of Akt could lead to the inactivation of tuberous sclerosis complex (TSC) 1/2, a critical factor of M2 polarisation and attenuation of M1 responses; in turn, mTORC1 would be further activated and increase the M1 phenotype [51]. The reduced activation of the Akt-mTOR pathway would result in a decreased M1 response [51]. The inactivation of p38 and ERK signalling would result from PGAM5 deletion in macrophages [51]. As a result, PGAM5 is a critical regulator of macrophage polarisation.

In addition to this, IL-38 is an anti-inflammatory cytokine associated with IA in the knee joint and shows higher serum levels in patients with RA compared to healthy controls [59]. This cytokine is produced by apoptotic cells and has a strong affinity with IL1RAPL1, the receptor for IL-38, to limit inflammatory macrophage activation [60]. IL-38 expression is increased by the inflammatory stimulation of cytokines, thereby implicating it in the pathogenesis of inflammation in the joint [59]. Human recombinant IL-38 was implicated in suppressing IL-6 and IL-1β in IA [61]. IL-38 pre-treatment results in the inhibition of synovial hyperplasia and decreased inflammatory cell infiltration and inflammatory factor levels [59]. IL-38 affects the glucose metabolism of M1 macrophages and its inhibition suppresses pro-inflammatory macrophages [59]. IL-38 can downregulate M1 pro-inflammatory markers, such as iNOS, TNFα and IL-6, through the inhibition of NF-κB and MAPK signalling and glucose uptake and glucose transporter 1 (GLUT1) expression [59]. This results in the downregulation of GLUT1 via IL-38-suppressed macrophage polarisation towards an M1 pro-inflammatory phenotype, and in turn, reduced synovial inflammation [59].

In several diseases, such as breast cancer and type 2 diabetes mellitus, adipokines promote inflammation and angiogenesis [2]. Fatty acid-binding protein 4 (FABP4) adipokine was found to be upregulated in M1 macrophages of patients with RA and murine models and its expression was reported to be proportional to RA severity [2,62]. It is secreted by macrophages and adipocytes and functions in regulating inflammation, promoting proliferation and angiogenesis and contributing to cancer and immunometabolic diseases [2]. It has been hypothesised that FABP4 upregulation takes place in RA chondrocytes due to excessive pro-inflammatory cytokine levels in the microenvironment [2,63]. Increased levels of FABP4 are regulated by the mTORC1 pathway to promote angiogenesis, synovitis and cartilage degeneration, thereby worsening the severity of the disease [2,64]. The mTORC1 pathways regulate synovial hyperplasia, inflammation and angiogenesis, and hence play an important role in RA progression [2]. The activation of mTORC1 induces the polarisation of macrophages towards the M1 phenotype and FABP4 secretion to promote further disease progression as seen in murine RA models. FABP4 is a main effector cytokine secreted by M1 macrophages which regulates human umbilical vein endothelial cell (HUVEC) tube formation through the activation of VEGFα expression [2]. FABP4 plays a role in synovial hyperplasia and infiltration by promoting HUVEC and FLS invasion and migration via the promotion of ERK1/2 of the MAPK pathway [2]. Recombinant FABP4 enhanced catabolism whilst suppressing anabolism in chondrocytes partly through the activation of the NF-κB pathway [2]. M1 macrophages expressing FABP4 can promote further inflammatory cytokine production by enhancing the phosphorylation of P65, a downstream effector of NF-κB, in HUVECs [2]. The inhibition of mTORC1 with rapamycin, an mTOR pathway inhibitor, via the ras homolog enriched in brain (Rheb1) in the myeloid lineage reduced the M1 macrophage expression of FABP4, resulting in an attenuation of RA development [2].

#### 2.1.3. Contribution to Inflammation

In patients with RA, M1 macrophages influence the occurrence of the Th1-mediated immune response in the inflammatory environment dominated by TLRs and IFN signalling [46]. This contribution is seen in the form of macrophage antigen presentation to naïve T cells, and in turn, a release of growth factors and cytokines. M1/Th1 cell crosstalk is mediated by MHC II and CD80/CD86 costimulatory molecules overexpressed in RA M1 macrophages [65]. This combination promotes the production of pro-inflammatory cytokines, chemokine factors and matrix metalloproteinases (MMPs), which when combined leads to erosion, osteoclastogenesis and progressive joint disease [66]. Osteoclastogenesis has been implicated to be enhanced by pro-inflammatory cytokines such as TNF-α, IL-1, IL-6 and IL-17 [67]. Therefore, an accumulation of inflammatory mediators due to M1 macrophages as a result of exaggerated immune activation contributes to enhanced OC formation [65]. The large production of pro-inflammatory mediators causes a drastic change in the synovial microenvironment by allowing the efficient activation of cytotoxic cells to take place. Cyclophilin A (CypA) induces an augmentation of the level of pro-inflammatory macrophages and cytokines present in the synovial fluid by increasing them and decreasing M2 macrophages [35]. This takes place via NF-κB-activating transcription which exacerbates RA and leads to a more aggressive disease course [35]. M1 macrophages are linked with the activation of the SAPK/MAPK and JAK/STAT pathways through their pro-inflammatory cytokines resulting in the promotion of proliferation and the survival of inflammatory macrophages [36]. The inflammatory process in RA is mediated and sustained by M1 macrophages [68]. Uncontrolled M1/Th1 activation in RA leads to organ damage via inflammation propagation [69].

There is no difference in the expression level of M2 macrophages between RA and PsA; however, PsA has a lower expression of M1 inflammatory cytokines compared to RA [30]. Newly recruited macrophages release similar levels of cytokines, contributing to inflammation in PsA and RA [34]. In PsA, M1 macrophages and T cells aggravate the disease via the production of inflammatory cytokines such as TNF-α, IL-23 and IL-17, which play a role in the occurrence and progression of PsA [31]. These inflammatory mediators stimulate the attraction of other inflammatory cells to the synovium and joint tissue, resulting in bone and cartilage deterioration. This leads to the breakdown of collagen, proteoglycans and gelatine, resulting in cartilage fibrillation and the apoptosis of chondrocytes (cartilage cells) [70]. Low paracrine levels of IFNγ or other soluble factors contribute to preferential macrophage polarisation towards the M2 phenotype in spondyloarthritis (SpA) synovitis, a destructive subtype of PsA [30]. TNF-α production induces angiogenesis, further driving the progression of joint inflammation [30]. Similarly to RA, PsA patients experience the development of erosion processes due to TNF-α, which induces NF-κB expression [71]. IL-17A, an IL-17 family member, stimulates pro-inflammatory cytokine production which has a catabolic role in osteoclastogenesis and bone metabolism [72]. Bone erosion is initiated by these cytokines due to their activating role in OC formation, which typically occurs away from the cartilage–pannus junction [73].

#### 2.1.4. Metabolism

M1 macrophages derive their energy from anaerobic processes, such as glycolysis, upon activation involving an increase in glucose uptake and pyruvate to lactate conversion [59]. GLUT1 induces glycolysis by activating NLRP3 and IL-1β in macrophages [59,74]. Respiratory chain activities are altered, thereby allowing for ROS production. LPS and IFNγ strongly induce glucose uptake with a suppression of fatty acid uptake and oxidation due to the activation of aerobic glycolysis [75]. This is due to the overexpression of GLUT1 [59,75]. In macrophages, GLUT1 is the main glucose transporter and drives macrophage inflammatory response by elevating the secretion of pro-inflammatory mediators in LPS-stimulated macrophages [59,75]. As a result of increased glucose production, GLUT1 expression is increased in knee IA [59]. Alterations in metabolic activity in M1 macrophages manifest as an enhancement in glycolysis in response to GLUT1 upregulation [50]. Therefore, the activation of LPS and IFNγ triggers a tricarboxylic acid (TCA), which generates citrate and succinate, crucial for fatty acid metabolism and the stabilisation of hypoxia-inducible factor (HIF)-1α [50,76]. HIF-1α plays a key role in maintaining cellular and systemic homeostasis in response to hypoxic conditions. It is expressed by synovial macrophages in RA to further promote macrophage activation and plays a key role in their commitment to glycolysis under normoxic conditions [50,76]. HIF-1α stabilisation leads to increased glycolytic flux in M1 macrophages. It is stabilised by succinate accumulation in LPS-induced macrophages, thereby promoting IL-1β expression [77]. This leads to the transcription of pro-inflammatory and glycolytic genes and epigenetic alterations [77]. Cytosolic citrate is essential for NADPH production and redox balance preservation. In this way, metabolic intermediates are not just an energy source for the cells but directly connect to macrophage phenotypes.

In addition to this, mRNA levels of IRG1, a TCA-cycle-related gene, are upregulated in synovial macrophages in the presence of SFs derived from patients with arthritis [50]. As a result, the TCA cycle is dysregulated. Glucose and glutamine uptake are increased by macrophages in the presence of SFs [50]. SFs promote the mRNA expression of cellular metabolism-related genes and metabolic reprogramming, necessary for immune responses in synovial macrophages [50]. As a result, M1 inflammatory markers, such as NOS2, IL-1β, CD86 and TNF, were upregulated by arthritis-derived SFs. This highlights that interactions mediated by secretory factors from arthritis-derived SFs have the ability to induce specific synovial macrophage phenotypes in a pathological synovial environment [50]. These phenotypes are long-lived with pro-inflammatory features that induce chronic inflammation in RA [50]. Macrophages have the capacity to influence SF phenotypes as demonstrated by Floudas et al., whereby TGF-β and IL-1β released by T cells and macrophages synergistically resulted in a highly glycolytic SF phenotype [29]. As a result, a shift in the metabolic profile of SFs is observed [29].

M1 macrophages also have an improved pentose phosphate pathway (PPP) which produces NADPH necessary for ROS production and arginine synthesis for catabolising arginine to NO [59]. Citrate-derived NADPH aids in the maintenance of redox balance by the production of NO and ROS inflammatory mediators and glutathione (GSH) antioxidants [78]. GSH prevents ROS-induced cell damage and preserves redox homeostasis. NO is considered to be a critical M1 metabolic regulator as it stimulates iron–sulphur-containing ETC clusters to become nitrosylated, and in turn, inhibits mitochondrial respiration and oxidative phosphorylation (OXPHOS) [79]. Therefore, classically activated macrophages have enhanced glycolytic metabolism and reduced mitochondrial activity [80]. Secretory factors released by SFs can alter synovial macrophage metabolic status by enhancing their glycolytic and oxidative metabolism [50]. This increases the lifespan of synovial macrophages in the inflammatory synovial microenvironment of IA [50].

### 2.2. M2 Macrophages

#### 2.2.1. Phenotype

Alternatively activated macrophages (M2) have an anti-inflammatory phenotype [46,51]. They are considered to have an alternative activation phenotype induced by IL-4 and IL-13 which opposes M1 polarisation driven by IFNγ. M2 macrophages are characterised by their ability to produce anti-inflammatory cytokines and chemokines which contribute to vasculogenesis, tissue remodelling, repair and wound healing [68]. M2 macrophages are also characterised by their ability to contain parasites and clear apoptotic cells [36]. They are known for their expression of mannose receptor-1 (CD206), macrophage scavenger receptors (CD204 and CD163) and Dectin whilst being associated with Th2 responses [32,33,51]. They produce immunomodulatory molecules such as TGF-β and IL-10 in response to stimulation by IL-4, IL-10, IL-13 and glucocorticoids [32,46,51]. M2 macrophages have high phagocytosis capacity with the ability to produce IL-10, ECM components and chemotactic and angiogenic factors [9]. M2 macrophages may also contribute to allergic inflammation, be cellular reservoirs of pathogens and aid the growth of tumour tissue [81]. These immune cells also play a role in organ morphogenesis, endocrine signalling and tissue turnover.

#### 2.2.2. Polarisation Conditions

TFs such as IRF-4, STAT6, c-MYC, PPAR-γ and KLF-4 are involved in M2 polarisation [82]. Monocytes differentiate into M2 macrophages when activated by macrophage colony-stimulating factor (M-CSF) and stimulated by immune complexes, complements and IL-4 [9,47]. IL-4 and IL-13 bind to the IL-4Rα receptor resulting in M2 polarisation [83]. IL-4Rα, in turn, activates JAK1 and JAK3, which lead to STAT6 activation and cause it to translocate to the nucleus and modulate interferon genes-mediated antiviral innate immune responses [83]. IL-10 induces an anti-inflammatory response by binding to its receptor, IL-10R [83]. This results in the autophosphorylation of the receptor and activation of STAT3 which, when bound to its promoter, can stimulate the expression of the suppressor of cytokine signalling 3 (SOCS3) which blocks the pro-inflammatory cytokine signalling pathway [83]. However, these cytokines can also induce the transcription of many M2 markers in other myeloid cells such as dendritic cells (DCs) [84]. M2 markers and the expression of IL-4/IL-13 are not exclusive hallmarks of M2 macrophages. The overexpression of some miRs such as miR-155 in the monocytes of patients with RA have been associated with defects of monocyte polarisation to anti-inflammatory macrophages [85].

Proteoglycan 4 (PRG4), a glycoprotein secreted by FLSs, is a boundary lubricant between cartilage surfaces preventing mitochondrial dysfunction [46]. It has been implicated in contributing to macrophage polarisation by influencing their responsiveness to stimulation. PRG4 interacts with CD44 to inhibit the nuclear translocation of NF-κB in SFs and reduces the expression of matrix-degrading enzymes and the proliferation of IL-1β-induced synoviocytes [46]. This glycoprotein suppresses TLR2 and TLR4 activation in IA via damage-associated molecular patterns (DAMPs) [46]. PRG4 recombination reduced the percentage of M1 macrophages and increased M2 macrophages [46]. Synovium lacking PRG4 displayed an age-dependent shift of macrophages towards the M1 phenotype, and in turn, a high M1/M2 ratio [46]. In the absence of PRG4, the anti-inflammatory activity of M2 macrophages is impaired as a reduction in IL-10 is observed and increased IL-1β and IL-6 followed by enhanced monocyte recruitment and differentiation into pro-inflammatory macrophages [46]. As a result, a lack of PRG4 leads to an increased M1 macrophage pool in the synovium and a further exacerbation of synovitis [46]. Macrophage depletion using liposomal clodronate reverses this synovial pathology. The glycoprotein employs direct anti-fibrotic, anti-inflammatory and anti-proliferative effects on SFs [86]. As a result, PRG4 maintains the homeostasis of synovial macrophages and the synovium.

#### 2.2.3. Contribution to Inflammation

Sustaining the M2 state of tissue-resident macrophages, such as adipose tissue macrophages and Kupffer cells, could diminish inflammatory mediator production and hence may be used as a therapeutic approach in the treatment of metabolic diseases [84]. The prolonged presence of tissue macrophages in the lining layer of the synovial membrane in healthy donors and patients with RA in remission hints that this population of macrophages may have a role in maintaining and reinstating synovial tissue homeostasis [11,46]. Macrophage subset disequilibrium is observed in patients with RA, with M1 macrophages favoured in active RA and M2 macrophages predominating during remission [39]. CD163, a haemoglobin scavenger soluble or membrane-bound receptor, is an IL-10 marker expressed by M2 macrophages and contributes to a local anti-inflammatory response by lowering haemoglobin levels and promoting inflammation-resolving haem metabolites [3]. Double-immunofluorescence staining has shown that intimal lining layer macrophages have an IL-10-like phenotype, whereas the synovial sublining macrophages have a heterogeneous phenotype [3]. Remodelling and repair are dynamic processes orchestrated by macrophages during ontogenesis and inflammation. M2 macrophages influence the Th2 response which determines the release of growth factors and cytokines involved in the anti-inflammatory process leading to a clinical remission of RA [69].

On the other hand, a recent study involving inflammatory cytokine association with IA using Mendelian randomization (MR) found that VEGF, IL-10, IL12p70 and IL-13 were associated with an increased risk of PsA [31]. These findings are consistent with other PsA studies [87,88]. CD163 expression is higher in SpA than RA synovitis, although the macrophage phenotype of both conditions is similar [3,30]. This suggests a potential bias towards the M2 phenotype in SpA during monocyte differentiation [3]. This contrasts with RA where these cytokines produced by M2 anti-inflammatory macrophages modulate anti-inflammatory activity. The MR analysis also indicated that genetic susceptibility to PsA promotes VEGF elevation [31]. VEGF has a potential role in PsA angiogenesis due to its implication in disease pathogenesis by regulating endothelial cell mitogenicity and vascular permeability [31].

The proline-serine-threonine phosphatase interacting protein 2 (PSTPIP2), known as the macrophage actin-associated tyrosine phosphorylated protein (MAYP), is mainly expressed by macrophages and is associated with autoinflammation [89]. Patients with progressive RA disease show significantly reduced levels of PSTPIP2 in synovial macrophages [89]. There is a positive correlation in patients with RA between the level of PSTPIP2 and frequency of CD206+ cells in peripheral blood [89]. PSTPIP2 negatively correlates with disease activity through macrophage polarisation towards the M2 phenotype [89]. The protein increases the number of M2 CD206+ macrophages and decreases the frequency of M1 CD86+ macrophages [89]. The PSTPIP2 regulation of macrophage polarisation is dependent on estrogen receptor (ER)-β [89]. A loss of ER-β results in the loss of PSTPIP2 activity. Hence, the anti-inflammatory effect of PSTPIP2 is synergic with M2 macrophages, as the PSTPIP2 cells form an immunological barrier in the synovial lining layer to induce the protection of the joint from the erosion of cartilage and bone [89]. During RA remission, the barrier is thick and compact in contrast to the active stage of disease in which the barrier is thin. Similar to CX3CR1+ macrophages, 85% of PSTPIP2 synovial macrophages expressed tight-junction proteins, such as zonula occludens 1 (ZO-1) and Claudin 5, reinstating their function in forming a barrier in the lining layer of the joint [11,89]. As a result, PSTPIP2 plays a role in the anti-inflammatory effect of M2 macrophages in IA.

#### 2.2.4. Metabolism

The M2 macrophage metabolic phenotype is significantly different from the M1 macrophages as they require a sustained supply of energy. This is supplied by oxidative glucose metabolism and fatty acid oxidation [50]. M2 macrophages produce ATP by driving pyruvate into the oxidative TCA cycle with OXPHOS. This is fuelled by mitochondrial fatty acid β-oxidation (FAO) and glutamine metabolism’s anaplerotic development of α-ketoglutarate [90]. M2 macrophages have an enhanced uptake of glutamine and fatty acids via CD36 upregulation leading to mitochondrial respiration [50]. The mitochondrial biogenesis and upregulation of FAO in macrophages polarised by IL-4/IL-13 is regulated by the mutual activity of STAT6, PGC-1β and peroxisome proliferative-activated receptors (PPARs) [91]. This is dependent on the induction of PPAR-γ to upregulate CD36 during the FAO process [91].

The major difference between M1 and M2 macrophage metabolism is related to L-arginine metabolism [92]. In M1 macrophages, L-arginine is metabolised to NO, whereas in the M2 phenotype, L-arginine metabolises to polyamines [44]. They catalyse L-arginine to urea and L-ornithine via Arg-1 induction [93]. It has been shown that L-arginine inhibits IA, bone loss and osteoclastogenesis by directly blocking TNF-α [94]. High levels of Arg-1 are characteristic of M2 macrophages as it plays a role in collagen synthesis, fibrosis, cell proliferation and other tissue remodelling functions [93]. Therefore, alternatively activated macrophages are characterised by high mitochondrial activity and OXPHOS [95].

### 2.3. Plasticity

M1 and M2 phenotypes represent extremes of their activation spectrum; however, it has been reported that markers for both phenotypes may coexist on the same cell [33,96]. A metabolic shift in fatty acid and glucose usage controls macrophage polarisation towards the M1 or M2 subtype, and in turn, their plasticity [97]. This is because macrophages are highly plastic cells and polarisation exists as a continuum with diverse intermediate phenotypes. This plasticity is achieved by a highly responsive epigenome determined by the occurrence of relevant DNA methylation changes [98]. This gives macrophages their heterogeneity and ability to change their functional phenotype in response to microenvironmental stimuli [46]. The phenotype of M1/M2 macrophages does not reflect the different phenotypic macrophage subsets [46]. M2 macrophages can express CD86 in response to TLR2 stimulation and possess a pro-inflammatory phenotype [46]. The plasticity of macrophages allows for monocytes to adopt the phenotype of tissue-resident macrophages, facilitating their recruitment in situations where tissue-resident macrophage replenishment is insufficient [99]. This is vital for the resolution of inflammation, tissue repair and remodelling [48]. Interaction with cells possessing progenitor or stem cell properties, such as mesenchymal stem cells (MSCs), is likely to be an important component of macrophages’ role in remodelling and repair [100]. MSCs attenuate the inflammatory environment by shifting macrophage phenotype from M1 to M2 with elevated levels of IL-4 and IL-13 and reduced TNFα and IL-6 cytokine levels. Elevated levels of anti-inflammatory cytokines activate macrophages via the IL-4Rα/Jak1/STAT signalling pathway to polarise into M2 macrophages [100].

In the synovium of patients with RA, macrophage populations show distinct phenotypes in different regions, which indicates a difference between infiltrating and tissue-resident macrophages. M2 macrophages display strong heterogeneity and plasticity in their phenotypes and can be influenced by various stimulating factors allowing for their further subdivision into M2a, M2b, M2c and M2d subtypes as seen in Figure 1 [40]. Each subtype demonstrates distinct characteristics and is crucial in different biological processes.

#### 2.3.1. M2a Macrophages

M2a macrophages are known for their wound healing activity and involvement in allergic reactions [101]. This macrophage subtype is activated through response to T cell-derived IL-4 and IL-13 cytokines via signalling through the IL-4Rα chain and the activation of M2-associated genes [102]. IL-4Rα signalling leads to STAT6 activation which in turn recruits PPARγ [102]. M2a macrophage differentiation is dependent on PPARγ for the expression of M2a differentiation genes [97,102]. M2a macrophages produce CD206 and fibronectin which operate in the enhancement of tissue repair via fibrotic lesion formation and ECM development [51]. They also express high levels of IL-10, TGF-β and CCL17 [103]. This subtype is marked by CD206+CD163−, reduced iNOS activity and a highly activated arginase pathway [97,104,105]. M2a macrophages function as anti-inflammatory mediators and play a role in tissue remodelling. An addition of recombinant PRG4 to TLR2-stimulated M2a macrophages reduces the expression and secretion of pro-inflammatory cytokines [46]. M2a macrophages are known for their capacity to suppress inflammation and aid in the stabilisation of angiogenesis [106]. It has been shown that M2a macrophages can undergo phenotypic and functional changes in the presence of IgG4 and repolarise towards an M2b-like phenotype [101]. IgG4 complexes strongly stimulate macrophages to produce IL-10 and CCL1, a ligand of CCR8 which is essential for the maintenance of M2b characteristics [101]. As a result, they shift from a wound healing state to an immunomodulatory phenotype, highlighting their plastic nature [101].

#### 2.3.2. M2b Macrophages

M2b macrophages are known as regulatory macrophages with immunomodulatory activity [54]. This subset is activated by responding to a combined exposure to IgG immune complexes and TLR agonists by expressing high levels of anti-inflammatory cytokines such as CCL1, IL-10 and TNF ligand superfamily member 14 (TNFSF14) [101,107]. They can also be induced by IL-1R agonists [54]. CCL1 is necessary for the maintenance of M2b macrophage properties [108]. Through the release of anti-inflammatory cytokines, such as IL-10 and IL-12, M2b macrophages play a role in Th2 activation and immunoregulation [54,105]. However, they also express pro-inflammatory cytokines such as IL-6, IL-1β and TNF-α [54]. As a result, M2b macrophages regulate the extent of the immune response and reaction. In contrast to M2a macrophages, M2b macrophages do not cause ECM deposition in IA [54]. In diseases such as pulmonary artery hypertension, M2b macrophages play a role in improving pulmonary remodelling and promoting apoptosis in pulmonary artery smooth muscle cells [54]. Apoptosis is induced via Bcl-2 family proteins and cleaved caspase-9 in M2b macrophages by regulating mitochondrial-dependent apoptotic signalling whereby the caspase promotes the release of cytochrome c and apoptosis-inducing factors [54]. M2b macrophages have a potential role in inducing tolerance to allergen immunotherapy through the secretion of CCL1 [101]. Hence, through the secretion of IL-10 and CCL1, M2b macrophages contribute to the development of an anti-inflammatory microenvironment [101].

#### 2.3.3. M2c Macrophages

M2c macrophages are involved in efferocytosis [97]. They respond to activation by glucocorticoids [51]. The M2c subset strongly exhibits anti-inflammatory activities against apoptotic cells by releasing high amounts of IL-10 and TGF-β and is marked by the expression of CD206 and CD163 [104,105]. CD163+ macrophages infiltrate sites of injury and inflammation during early stages of wound healing [106]. IL-10 activates M2c macrophages through the activation of STAT3 via IL-10R whilst TGF-β provides M2c macrophages with regenerative activity for cartilage and bone regeneration [108,109]. Matrix remodelling is an important aspect of restoring healthy tissue to the inflammatory site [106]. This subset upregulates matrix remodelling enzymes, permitting fibrosis regulation [106].

M2c macrophages are important phagocytes for the clearance of apoptotic cells and cell debris due to their high phagocytic capacity for apoptotic cells in vitro and upregulation of phagocytic genes such as CD163 and MARCO [106]. Their release of IL-10 and over-expression of Mer receptor tyrosine kinase (MerTK) facilitates efficient phagocytosis [110]. Studies have also shown that M2c macrophages are involved in angiogenesis through the upregulation of genes such as SRPX2 and VCAN, which are involved in the process [106,111]. Therefore, M2c macrophages function in immunosuppression, phagocytosis, matrix deposition and tissue remodelling [105].

#### 2.3.4. M2d Macrophages

M2d macrophages are induced by IL-6, TLR and A2 adenosine receptor agonists [84]. The activation of adenosine receptors is followed by the suppression of TLR-dependent expression of pro-inflammatory cytokines and induction of the secretion of anti-inflammatory cytokines, such as IL-10 and TGF-β, and vascular endothelial growth factor (VEGF) [112]. The IL-10 signalling pathway is activated in M2d macrophages, thereby promoting mucosal and epithelium healing and inflammation resolution [113]. IL-10 inhibits the excessive pro-inflammatory response by inhibiting the MAPK pathway. It also suppresses IL-1, IL-6, IL-8, GM-CSF and TNF-α synthesis, which are known pro-inflammatory mediators [113].

M2d macrophages have proangiogenic properties with features of TAMs for tumour progression by supporting the tumour microenvironment and metastasis [91,112,113]. Pro-inflammatory cytokines such as IL-6, IL-10 and VEGF are present in the tumour microenvironment and contribute to the accumulation of M2d macrophages by preventing the differentiation of monocytes into DCs [114]. It was recently shown that there is potential communication between M1 and M2d macrophages via VEGF and the CCL3-CCR1 signalling pathway [113]. This pathway has been associated with an increased promotion of polarisation towards the M1 phenotype. VEGFA is secreted by both M2d and M1 macrophages [113]. VEGF signalling exacerbates inflammation and tumour growth. Hence, M2d macrophages have a dual role of mediation of inflammation resolution and angiogenesis.

#### 2.3.5. Repolarisation

It has been hypothesised that the phenotype of polarised M1 and M2 macrophages can be reversed to a certain extent *in vitro* and *in vivo*. However, it is unclear whether the mechanism of these switches involves the re-education of cells *in situ* or the recruitment of circulating precursors. After activation, macrophages maintain their plasticity and can shift from their functional phenotype to another depending on their microenvironment by changing their activation state. In this way, an M2 macrophage can repolarise to M1 in response to M1 stimulation such as IFNγ [115]. TLR2 stimulation also drives M2 macrophages to secrete pro-inflammatory cytokines and impairs their anti-inflammatory activity [116]. This stimulation induces the generation of chimeric M1/M2 macrophage subsets without major changes in their surface marker profile; however, the cytokines and chemokines they secrete are different and reflect their hybrid-plastic phenotype [116]. The activation of TLR agonists in the presence of an adenosine A_2A_ receptor (A_2A_R) leads to macrophage repolarisation from the M1 phenotype towards the M2d phenotype [112]. Under inflammatory conditions such as IA, the classical M1/M2 macrophage model based on surface marker expression does not apply to macrophage function [116]. As a result, the intermediate macrophage phenotypes show a co-expression of both M1 and M2 markers [117]. This highlights the significance of the native cytokine milieu in directing macrophage polarisation.

In a recent study by Choi et al., the effects of pyropia yezoensis glycoprotein (PYGP) on M1 and M2 polarisation and the potential for repolarisation were examined. LPS-stimulated M1 macrophages treated with PYGP showed a reduced production of M1 markers and instead increased M2 marker expression [44]. An increase in arginase 1, peroxisome proliferator-activated receptor γ (PPAR-γ), RETNLB, IL-10, CD163 and CD206 were observed [44]. PPAR ligation inhibits pro-inflammatory cytokine and NOS2 expression [118]. STAT3 and STAT6 TFs were phosphorylated post-PYGP treatment [44]. These TFs are known to induce M2 macrophage activation and inhibit inflammation. However, their silencing using siRNA decreased M2 marker gene expression in macrophages despite PYGP treatment. This suggested that PYGP applied an anti-inflammatory effect on M1 macrophages by regulating the M1 to M2 phenotypic switch via STAT3 and STAT6 phosphorylation [44]. This indicates that macrophages can repolarise from an M1 to M2 phenotype, and this phenomenon could be utilised as a potential therapeutic approach to IA diseases.

In addition, ageing causes a shift in macrophage polarisation patterns from M2 to M1, further highlighting their temporal plasticity [119]. As a result, it is possible that changes in the microenvironment may lead to a macrophage phenotype switch from anti-inflammatory to pro-inflammatory and an increase in pro-inflammatory cytokines and immune cells [119]. One such example is related to age-dependent changes with neuronal loss and a degeneration of the enteric nervous system (ENS). The ENS undergoes neuronal loss and degenerative changes with age. This phenotypic shift associates with a neuronal response to inflammatory signals, increased apoptosis, tissue damage, a loss of enteric neurons and enteric neural stem cells [119]. Muscularis macrophages residing close to the enteric ganglia maintain the neuromuscular function of the gut by precise crosstalk with the enteric neurons [119]. The implication of macrophage repolarisation with age may contribute to IA pathogenesis in older populations.

## 3. The Role of Macrophages in the Pathogenesis of IA

### 3.1. Macrophage Correlation with Disease Progression

Macrophages play a crucial role in RA pathogenesis and prognosis due to their wide range of immunomodulatory, inflammatory and tissue-repairing functions. In a normal synovial membrane, only a few macrophages are present within the stromal tissue, which primarily is comprised of sparse blood vessels and FLSs [120]. When clinical symptoms of joint swelling are observed, significant changes are seen in the synovium. A thickening of the lining layer is observed due to increased macrophages and FLSs. The cellular infiltration of innate and adaptive immune cells, such as macrophages, DCs, natural killer cells and T and B lymphocytes, takes place. Thus, a high presence of macrophages in the joints is a feature of inflamed areas and an early indicator of active disease [121]. An absence of ACPAs in RA (ACPA-negative patients) has prognostic value for drug-free remission [39]. As a hallmark of inflammation, an abundance of M1 macrophages in RA synovitis is reflective of disease activity and hence, their depletion at the target organ level is a good biomarker for therapeutic response [122].

In a recent study by Alivernini et al., single-cell RNA sequencing (scRNA-seq) has shown that active RA patients have heterogenous SFs and macrophages which may have important implications for therapies aimed at the modulation of inflammation or tissue repair [39]. Fluorescence-activated cell sorting (FACS) was carried out on synovial tissue macrophage (STM) populations and gated on CD45, CD64, CD11b and HLA-DR expression with an evaluation of MerTK, CD163, CD206, TREM2 and FOLR2 expression [39]. Transcriptomic profiles of scRNA-seq of STMs and whole synovial tissue were compared for RA groups (treatment naïve, resistant and in remission) and healthy controls [39]. A unique transcriptomic profile was observed in the remission group, enriched for negative regulators of inflammation [39,89]. Healthy STMs were found to be mainly MerTK+CD206+, and were also significantly increased in patients in remission compared to those with active disease [39]. These STMs were composed of TREM2+ and FOLR2+LYVE1+ clusters which possess complementary roles in controlling local immune response and homeostasis [39]. In addition to this, TREM2+MerTK+ subpopulations are a potential source of resolvins, which induce a repair response in FLSs and have a reduced likelihood of flare after treatment cessation, thereby promoting inflammation resolution [39,123]. Hence, sustained remission is an active process maintained by tissue-resident subpopulations of MerTK+CD206+ STMs commanding SFs to reinstate and maintain homeostasis at the inflammatory site [39]. This reinforced the idea of patient stratification based on transcriptomic profiles and their use for the prediction of patient-specific response to treatment [124].

A recent study by Li et al. defined STM populations via the scRNA-seq of patients with IA who had knee replacement surgery [45]. Physical interference is typically carried out after the failure of nonsurgical therapeutics, hence the STM populations from these patients represented end-stage and drug-modified histopathological profiles. A higher proportion of CD206+ M2 macrophages were found in the synovium of patients with RA that clustered in the lining layer of the synovium. Trajectory analysis showed that cells predisposed to polarise into M1 macrophages highly expressed inflammatory genes such as IL1B, CCL20 and NFKM1, whereas M2 macrophages expressed CCL2, NRP1 and IGF1 anti-inflammatory genes [45]. The previous findings of Alivernini et al. were reinforced in this study, as SPP1 and MerTK were predominantly expressed by M1 and M2 macrophages, respectively [45]. TREM2 was predominantly expressed in non-polarised macrophages. STMs from seropositive and seronegative patients with RA colocalised with CD55+ lining, high HLA-DRA and CD34+ sublining fibroblasts. Transcriptomic profiling provides insight into macrophage polarisation and can be used to correlate macrophage phenotypes with disease progression.

#### 3.1.1. M1 Macrophages in IA Pathogenesis

In the early stages of RA, pro-inflammatory cytokines produced by M1 macrophages contribute to the infiltration and activation of peripheral blood MDMs, which leads to the promotion of inflammation in the synovium [68]. The depletion and low numbers of M2 macrophages inhibit the formation of highly vascularised cellular granulation tissue and scar tissue, thereby allowing for further bone damage and erosion to take place [125]. ACPA-positive patients have an increased risk of developing bone erosion, and in turn, a worse disease prognosis [126]. This bone resorption process takes place either by an immune complex-mediated activation of M1 macrophages that secrete pro-inflammatory cytokines promoting OC differentiation or direct recognition of citrullinated proteins by OC precursor cells leading to OC generation [92,127,128]. The imbalance in the M1/M2 macrophage phenotype disrupts the normal homeostasis between osteoclasts and osteoblasts, leading to the loss of bone homeostasis and remodelling [129]. The indirect route of OC formation involves the ability of ACPAs to form immune complexes with their target citrullinated proteins [92,127]. Fc receptors on macrophage surfaces direct the induction of TNFα, and as a result, receptor activator of NF-κB ligand (RANKL) expression, which in turn promotes osteoclastogenesis [92,127]. OC activation and differentiation by ACPAs have also been related to the development of joint pain [130]. The synovial tissue responds to the inflammatory signals during disease progression by undergoing changes such as neovascularization, inner-layer hyperplasia, inflammatory and mesenchymal cell infiltration and pannus formation [65]. These processes collectively result in the destruction of cartilage in the joints. Inflammatory macrophages are also involved in the turnover of connective tissue and articular surface erosion due to their production of MMPs which contribute to the progression of early inflammation towards chronic IA [131]. RA patients with M1/M2 ratios > 1, with relatively more M1 monocytes, had higher CRP and ESR than RA patients with M1/M2 ratios ≤ 1 which thereby correlated with a worse clinical course of disease [69]. Hence, M1 macrophages are correlated with worse disease progression due to their aggravating effects.

#### 3.1.2. M2 Macrophages in IA Pathogenesis

Alternatively activated and STMs are associated with maintaining joint homeostasis during remission [39]. M2 macrophages contribute to inflammation resolution via the phagocytosis of debris and apoptotic neutrophils and the production of anti-inflammatory mediators and are a source of lipid mediators [132]. Lipid mediators such as lipoxins have anti-inflammatory and pro-resolving characteristics. Macrophages undergo dynamic changes during phases of wound healing. During the early stages of tissue repair, infiltrating macrophages have an M2 phenotype. In a peritoneal inflammation model, resolution-phase macrophages express a unique plastic M1/M2 phenotype in which cAMP was essential for downregulating M1 activation [132]. The inactivation of M2 macrophages correlates with impaired tissue remodelling and homeostasis [36]. Therefore, the presence of M2 macrophages correlates with better disease progression due to their anti-inflammatory properties skewing synovial inflammation towards remission.

### 3.2. Predisposition of RA Patient Monocytes to Differentiate into Pro-Inflammatory Macrophages

Macrophages, especially the M1 phenotype, are crucial contributors to IA pathogenesis, releasing chemokines, cytokines and inflammatory molecules which promote inflammation and lead to tissue damage in affected joints [68]. In RA, TLR agonists and Fc receptor engagement activate innate immunity in the synovium, thereby initiating inflammation. Monocytes and synovial macrophages highly express TLR2, TLR4, TLR5, TLR7 and TLR8 promoting inflammation and cytokine production [33,133]. In addition to this, when tissue injury takes place, monocytes in circulation are recruited and migrate to the synovium in a CCR2-dependent manner where they become a source of synovial macrophages [109,134]. Bone marrow-derived and resident macrophages proliferate independently from one another with limited proliferative activity shown in resident macrophages during IA [109,134]. These macrophages maintain their joint integrity and restrain the progression of inflammation [11,109,135]. On the other hand, tissue and joint infiltrating macrophages initiate the inflammatory process and drive IA pathogenesis. These cells have a predisposition to differentiate into pro-inflammatory M1 macrophages which are known to exacerbate inflammatory processes and result in worse disease progression [11,109,135].

Patients with RA with defective bone marrow monocytes polarised into M2 macrophages have decreased CD206, YM-1 and IL-10 levels [85]. This defect is associated with an increase in miR-155-5p in monocytes and M2 macrophages which allows the progression of the inflammatory response [85]. miRs are implicated as mediators of disease pathology through a coordinated regulation of molecular effector pathways [136]. In a recent study by Narayan et al., RA patient monocyte differentiation into pro-inflammatory macrophages has been correlated with the downregulation of a mitochondrial translocator protein (TSPO). The study demonstrated that the activation of pro-inflammatory MDMs from healthy and RA patients downregulated TSPO, which was associated with a reduction in vital components of the cholesterol efflux pathway [137]. RA monocytes with downregulated TSPO may be a novel pathogenic mechanism in RA which predisposes patients to an inflammatory macrophage phenotype and in turn promotes chronic inflammation [137].

Folkersen et al. created the COMBINE biobank, a collection of multi-omics data including RNA-seq, DNA and protein biomarkers, from 185 RA patients for patient stratification and the prediction of clinical response to anti-TNF therapy [138]. Transcriptomic analysis demonstrated that AKAP9, CX3CR1, SLC2A3, SORBS3, CYP4F12, MUSTN, TBC1D8 and C21orf58 gene expression could be used as stratification biomarkers at *p* < 0.05 [138]. The potential prediction of CXCL13 levels on disease course was observed linking it to disease severity [138,139,140,141]. It has been previously described as a target engagement biomarker for germinal centre activity in the phase 3 Monarch study in RA for both anti-TNF (adalimumab) and anti-IL-6 (sarilumab) [141,142]. CXCL13 plays an important role in establishing the adaptive immune response by attracting B cells which aid in the generation of antibodies and local inflammation [140]. The chemokine is associated with involvement in the joint in early RA and acts as a route in disease progression [140]. Studies suggest that high CXCL13 serum protein levels in patients with RA are associated with an improved chance of sustained remission after 2 years [138,140]. High concentrations in plasma are indicative of recent inflammation onset [138]. These patients may respond better to early aggressive treatment [138], whereas patients with RA with high CXCL13 gene expression levels in the synovium are associated with a worse disease course and hence a different treatment approach would be needed [138]. Other studies have suggested that the chemokine has pro-inflammatory activity and is secreted by M1 macrophages [143]. It has been shown that M1 macrophages can induce endothelial hyperpermeability and promote p38 phosphorylation in sepsis via PPAR-γ inhibition and increased CXCL13 production [143]. This, in turn, would lead to increased inflammatory infiltration. Hence, further research is needed to understand the mechanism of CXCL13 in IA diseases.

### 3.3. Potential Impairment in Phagocytosis

M2 macrophages display high phagocytic capacity which aids inflammation resolution by inhibiting the production of pro-inflammatory cytokines due to TGF-β and IL-10 release [144]. Phagocytic capacity is provided by the presence of lysosomes and pinocytic structures with digestive enzymes [100]. This inhibition involves an autocrine or paracrine secretion of TGF-β which inhibits further monocyte and macrophage secretion [144]. However, ablating strategies aiming to reduce pro-inflammatory macrophages in the joints of RA patients have had variable success. Murine studies have shown that depletion is linked to adverse effects such as reduced collagen deposition, immunosuppression, infection and impaired phagocytosis, angiogenesis and wound healing [145,146]. Chronic inflammation also has the potential to impair macrophages’ ability to phagocytose apoptotic cells via efferocytosis and cell debris [147]. This is commonly seen in tobacco-smoking individuals due to the additional neutrophil burden and impaired alveolar macrophages [147]. This prevents effective pathogen clearance and inflammation resolution from taking place and contrarily promotes the pro-inflammatory environment. The dysfunctional phagocytosis capability of macrophages results in a build-up of apoptotic material in the synovial microenvironment leading to increased cellular infiltration, and in turn, inflammation. Viral infection can impair and lead to the dysfunction of M1 and M2 macrophage polarisation [36]. This has been observed in patients with chronic hepatitis C virus (HCV) infection due to the core protein of HCV inhibiting the polarisation pathways of MDMs via TLR2 signalling [36,148]. As a result, this would exacerbate IA further and impair subsequent phagocytosis by macrophages.

### 3.4. Protective Role of CX3CR1 Macrophages

As described by Culemann et al., 2019, barrier-forming CX3CR1+ lining macrophages form a dynamic membrane-like structure around the synovial cavity, providing a protective barrier for the joint [11]. These macrophages physically seclude the joint, maintain their number independent of blood monocytes and constitute 40% of the total synovial macrophages under steady-state conditions [11]. At the onset of RA, CX3CR1+ macrophages change their spatial orientation and morphology; however, their position is maintained and they neither proliferate nor change in number [11]. On the contrary, CX3CR1− macrophages rapidly proliferate and contribute to the pool of CX3CR1+ macrophages [11]. scRNA-seq revealed heterogeneity among the CX3CR1− interstitial macrophages. One population expressed high levels of mRNA encoding MHCII and aquaporin, and another population was characterised by *RETNLA*, which encodes RELM-α, *CD163* expression which is implicated in M2 macrophage activation [11]. Despite an increasingly inflammatory microenvironment, CX3CR1+ macrophages stably maintain their immune-regulatory phenotype, such as the expression of *TREM2*, and high levels of receptors, such as *AXL* and *MFGE8*, mediating apoptotic cell clearance [11]. CX3CR1+ macrophages continue to express mRNAs encoding tight-junction proteins and genes involved in planar cell polarity. Due to their protective role, definite adherens and tight junctions, desmosomes and prominent cellular interdigitations can be found at the cell–cell border of these macrophages [11]. However, as IA progresses with age, the CX3CR1+ subset can diminish, signalling a compromised endogenous anti-inflammatory role of synovial macrophages [46].

Additionally, Culemann et al. studied the role of these tight-junction-expressing CX3CR1+ macrophages during RA by depleting these macrophages in mice using diphtheria toxin [11]. Tight junctions between synovial lining macrophages rapidly disintegrated leading to the loss of the synovial barrier even in healthy mice which correlates to the physical density changing in this macrophage network during inflammation onset and resolution [11]. This breakdown takes place after autoantibody-containing immune complexes deposition; these are immediately ingested by CX3CR1+ lining macrophages. The disintegration of tight junctions is independent of inflammatory myeloid cell recruitment and results from an initial immune-complex-mediated activation of the lining macrophages [11]. This results in disruption to the synovial barrier function and an early and exacerbated onset of IA and accelerated polymorphonuclear leucocyte influx.

## 4. Therapeutic Approaches Targeting Macrophages for the Management of IA

Treatment of RA and PsA has been revolutionised by the implementation of aggressive treatment strategies such as Treat to Target, the introduction of Early Inflammatory Arthritis Clinics and the availability of therapies directed against soluble mediators (TNF inhibitors and IL6 blockers), immune cells (B cells) and intracellular signalling pathways (JAK inhibitors) [149]. However, the high cost and potential side effects of biological therapies in particular underline the need to identify predictive markers of response and new therapies to ensure optimum patient outcomes [150]. Approximately 50% of patients do not respond to individual agents with periods of active disease needing to be controlled with glucocorticoids [151]. Both high disease activity and high dose glucocorticoid use are associated with increased morbidity and mortality in patients [152]. In as many as 20% of cases, patients are resistant to all current medications, highlighting the unmet need for patients with inflammatory arthritis [150]. The molecular mechanisms that underpin failure to respond to approved therapies remain to be elucidated. Case studies have highlighted the importance of synovial macrophages as a predictor of response to anti-TNF in RA [41,153], whilst a recent study reported that response to Tocilizumab (IL-6 inhibitor) is associated with the presence of myeloid cells in synovial B cell-poor subjects [154]. Nonetheless, validated biomarkers able to predict response to specific agents in RA and PsA are lacking, although the above studies suggest that, given the highly heterogeneous nature of both diseases, it is likely that different pathways are active in individual patients [155]. Non-response appears to be characterised by a fibroid pauci-immune pathotype associated with a scanty immune cell infiltrate with the prevalence of stroll cells [154]. Given the key role of macrophages in disease pathogeneses and their presence in the synovium of both RA and PsA patients, studies that enhance the knowledge of their role and polarisation in disease offer the potential to identify biomarkers of response and new targeted therapies improving patient outcomes.

The number of macrophages at the synovium significantly correlates with disease severity [96]. In addition to this, no treatment targeting macrophages specifically is currently used in clinics [96]. Hence, an approach to target the depletion of inflammatory macrophages whilst sparing other subsets and re-establishing the macrophage balance may be an effective therapeutic approach for the management of IA [96].

### 4.1. Targeting M1 Macrophages

DMARDs have been commonly used to combat inflammatory mediators such as those expressed by M1 macrophages. MTX, a csDMARD, is a crucial first-line treatment for RA patients and is commonly used in combination with other drugs [156]. The combination of MTX and Shikonin has been demonstrated to inhibit M1-type macrophage polarisation and promote M2-type polarisation [157]. This change in macrophage polarisation may contribute to the benefits of these therapies. MTX effectively reduces disease activity and slows down joint damage with a significant percentage of early RA patients achieving remission or low disease activity [158]. RA patients demonstrate a higher reduction in disease activity measures than PsA patients [158]. However, approximately 30% of RA patients do not respond to MTX [138]. The effectiveness of MTX in PsA patients has remained controversial, as clinical trials have failed to reach significant improvements in comparison to the placebo [158].

A bispecific targeting of TNF and IL-6 cytokine production by FLSs, macrophages and T cells was identified as a promising therapeutic approach to treating RA patients [141]. Anti-TNF and anti-IL-6 antibodies were combined in IA murine models and exhibited sustained long-term remission alongside improved histology and bone remodelling [141]. An anti-TNF/IL-6 bispecific nanobody, Compound 1, was developed with similar potencies against IL-6 and TNF and simultaneous binding to both cytokines. The Biesemann group demonstrated that Compound 1 or a full dose combination of anti-TNF/IL-6 completely inhibited CXCL13 induction compared to partial inhibition by FDA-approved adalimumab and sarilumab [141]. The bispecific nanobody is superior to monospecific antibodies due to its ability to bind both cytokines and additively inhibits CXCL13 [141]. A further evaluation of the anti-TNF/IL-6 antibodies’ efficacy in clinical studies is required.

The JAK inhibitor Baricitinib has been FDA approved for treating RA whereby it prevents the activation of the STAT pathway and inhibits the transcriptional initiation of effector genes [96]. As a result, Baricitinib prevents the inflammatory and autoimmune reactions associated with RA such as the secretion of IFNγ [96]. Studies have supported the effects of JAK inhibitors on macrophage phenotypes in RA whereby the metabolic profile in the site of inflammation is shifted from M1 to M2 and metabolism is reprogrammed towards OXPHOS [96,159]. The use of ERK1 and Notch1 inhibitors is a new potential route for altering M1 macrophages. A knockout of ERK1 results in the suppression of M1 macrophage proliferation [96]. Notch1 signalling regulates M1 macrophage fate via direct transcriptional and indirect metabolic regulation [96]. Hence, pairing ERK1 with Notch1 would result in the death of M1 macrophages [96]. The use of ERK1 and Notch1 inhibitors has been tested separately in IA mouse models and shown to reduce inflammation [96]. The therapeutic value of co-targeting ERK1 and Notch1 has yet to be demonstrated for RA but was shown effective in cancer in which Notch1 targeting enhanced ERK1 inhibitors’ efficacy in cancer patients [96]. Hence, the co-targeting of ERK1 and Notch1 could be a promising therapeutic strategy for IA patients.

In a recent study, the use of BMS309403, an FABP4 inhibitor, and anagliptin, an inhibitor of dipeptidyl peptidase 4, have been shown to inhibit the expression of FABP4 in serum and synovial M1 macrophages in mice [2]. BMS309403 effectively reverses the increased proportion of M1 macrophages. A reduction in FABP4 levels led to the alleviation of the progression of RA [2]. This prevented further RA progression through the inhibition of synovitis, reduction in SFs, suppression of the angiogenesis of H-type vessels and reduced invasive capacity of FLSs in the synovium [2]. BMS309403 and/or anagliptin demonstrated downregulated MMP13 expression and OCs. These finding indicate a potential route of RA treatment by targeting M1 macrophages secreting FABP4.

### 4.2. Targeting M2 Macrophages

M2 macrophages are necessary for the resolution of inflammation in IA. Studies have shown the potential use of bDMARDs for inflammation resolution through their effect on macrophage polarisation and their ability to repolarise M1 macrophage into the M2 phenotype. Anti-TNF-α agents have also demonstrated the ability to induce polarisation towards an alternative anti-inflammatory phenotype in macrophages [160]. Anti-TNF agents such as etanercept and adalimumab favour anti-inflammatory M2 surface markers such as CD163 and MerTK whilst decreasing the expression of CD40 and CD80 pro-inflammatory M1 markers [160]. Anti-TNF-α agents shift macrophage polarisation towards the M2c macrophage subset through the induction of CD163 and MerTK expression which in turn leads to increased IL-10 secretion [161]. IL-10 is responsible for negative feedback control of inflammation by signalling through the Jak/STAT pathway involving SOCS3 and growth arrest specific 6 (GAS6) [160]. GAS6 inhibits monocytes from secreting IL-1, IL-6 and TNF [160]. As a result, anti-TNF agents inhibit macrophages’ inflammatory functions and favour the resolution of inflammation through a modulation of macrophage polarisation in an IL-10/STAT3-dependent manner [160].

Treatment with the CTLA4-Ig (Abatacept) bDMARD has been shown to induce a shift in macrophage polarisation, based on their plasticity, from a pro-inflammatory M1 phenotype to an anti-inflammatory M2 phenotype in both MDMs from healthy and active RA patients [161]. RA patients who do not respond sufficiently to bDMARDs or MTX have shown reduced disease activity after abatacept treatment and this has higher safety compared to alternative treatments such as Upadacitinib, a JAK inhibitor, as seen in the SELECT-CHOICE trial [162]. Specifically, the treatment led to a downregulation of M1 markers (TLR4, CD80 and CD86) and an upregulation of M2 markers (CD163, CD204 and CD206), thereby proposing a heightened induction of the macrophages’ phagocytic activity [161]. These effects were observed at different time points with significant changes after 12 h of treatment [161]. An increased expression of CD163 via abatacept treatment can promote the macrophage maturation process towards the M2 phenotype, restoring the M1/M2 disequilibrium associated with the chronic inflammation of RA [161]. Abatacept induces a shift from M1 to all subsets of M2 macrophages [160]. This is due to the treatment’s capacity to induce CD163 and MerTK together with CD204 and CD206 which mediate IL-4 secretion [161]. RA patients in clinical remission demonstrate elevated levels of CD163, CD206 and MerTK [39]. Hence, utilising treatment such as abatacept in IA to restore the M1/M2 imbalance and express anti-inflammatory mediators is a vital aspect of the resolution of inflammation and amelioration of disease progression.

Through the use of liposomal encapsulation, it was shown to be possible to deliver an antagomiR in monocytes and macrophages to the site of inflammation whilst avoiding systemic side effects [85]. Aberrant miR-155 is induced by TLR stimulation in macrophages, enhances inflammatory cytokine production and has been found to be elevated in the synovial fluid and tissue of RA patients [163]. miR155-5p expression is higher in M1 macrophages [163]. Hence, silencing miR-155-5p would allow for monocytes to polarise into M2 macrophages [85]. Paoletti et al. developed an antagomir-155-5p encapsulated in PEG liposomes which targeted the polarisation of monocytes into M2 macrophages in RA models. Once the liposome is injected into blood circulation, it passively extravasates and accumulates at the inflammatory site [85]. The antagomir-155-5p-encapsulated PEG liposome has improved drug distribution resulting in increased drug efficacy, prolonged lifetime and reduced toxicity and side effects affecting other immune cells [85]. The anatgomiR-155-5p acts at the stage of blood-circulating monocytes by reorientating them to differentiate into M2 macrophages [85]. This treatment was able to reduce immune cell infiltration, restore normal monocyte polarisation, decrease the expression of mRNA targets of miR-155-5p, such as SOCS1 and CEBPβ, and increase CD206+ and CD163+ macrophages [85]. Monocyte polarisation was restored to the M2 phenotype with an increased expression of YM-1 and CD206 and IL-10 secretion [85]. As a result, anti-inflammatory activity takes place in an attempt to restore synovial homeostasis. However, the treatment did not impact CX3CR1+ macrophages as there was no restoration of CX3CR1+MerTK+ lining layer macrophages [85]. This injection of antagomir-155-5p is a useful therapeutic approach in delivering small RNAs to monocytes and macrophages and directing them into the anti-inflammatory phenotype in order to alleviate joint inflammation in RA in a safe and specific manner [85].

### 4.3. Targeting Plasticity

Therapeutically reprogramming macrophages from the M1 to the M2 state can inhibit subchondral bone destruction and reduce joint damage linked to adjuvant-induced IA. Li et al. demonstrated a new approach in targeting the plasticity of macrophages in the treatment of IA using an RA mouse model. Plasmid DNA encoding IL-10 (IL-10 pDNA) was encapsulated with a chemotherapeutic drug, betamethasone sodium phosphate (BSP), in a biomimetic M2 exosome (M2 Exo) vector derived from M2 macrophages [164]. This approach used Exo as an endogenous drug carrier with inflammation targeting and anti-inflammatory properties to increase IL-10 secretion and suppress pro-inflammatory cytokine production [164]. M2 Exo/pDNA/BSP effectively polarised M1 macrophages into M2 macrophages with a strong inhibition of disease progression without noticeable off-target side effects [164]. The anti-inflammatory effects of the treatment are due to the synergetic effect of the gene and drug [164]. IL-10 inhibits NF-κB activation and promotes the repolarisation of macrophages towards an anti-inflammatory phenotype via positive feedback [160,164]. BSP binds the glucocorticoid receptor in the target cytoplasm and thereby inhibits pro-inflammatory cytokine transcription and cytokine-mediated inflammation. The study showed that the circulation of M2 Exo with the codelivery of pDNA and BSP could significantly improve the therapeutic efficacy in RA with reduced adverse effects and negligible toxicity [164].

Imatinib, a tyrosine kinase inhibitor, has shown the ability to exert positive effects of lessening the clinical symptoms of patients with RA [165]. It has previously been used to stabilise the formation of tight junctions at the blood–brain barrier and shown to stabilise the formation of tight junctions by CX3CR1+ lining macrophages and in turn interfere with the onset of IA [11,166]. This did not affect arthritis onset but accelerated the resolution of inflammation [11]. Imatinib attenuates RA-FLS inflammation by inhibiting proliferation and promoting apoptosis, ultimately minimising the damage caused by chronic inflammation [165]. It operates by suppressing CSF1R expression which previously promoted proliferation, inhibited apoptosis and accelerated the cell cycle [165]. CSF1R is responsible for the differentiation and survival of the monocyte/macrophage cell lineage [165]. The inhibitor could be used to reinstate CX3CR1 levels in the synovium.

Recently, M2a macrophages have been utilised in IA treatment to evoke anti-inflammatory and regenerative processes [167]. Stable macrophages were constructed from rat bone marrow in which anti-inflammatory and pro-regenerative M2a polarity (L-M2a) was locked via the knockout of TNFR1 and IL-4 overexpression [167]. L-M2a macrophages were engineered using CRISPR-Cas9 genome editing with over 60% efficacy of TNFR1 knockout. The macrophages maintained a stable anti-inflammatory phenotype and resisted repolarising to a pro-inflammatory phenotype in an IA microenvironment [167]. A high expression of IL-4, IL-10, TGF-β and arginase 1 was observed by L-M2a macrophages. However, L-M2a stability decreased when stimulated with LPS and IFNγ. The intra-articular injection of L-M2a macrophages delayed the process of IA by maintaining high CD206 levels. They also inhibited pro-inflammatory cytokine production via crosstalk with IA-FLSs and evoked an anti-degenerative effect in crosstalk with senescent chondrocytes [167]. L-M2a alleviated cellular senescence, apoptosis and the degenerative phenotype of IA by restoring homeostasis and promoting cartilage regeneration whilst maintaining regenerative capacity in the IA microenvironment [167]. Despite the promising results, further testing and clinical trials are necessary for using the cell transplantation of L-M2a and macrophage specific treatments in clinics.

## 5. Conclusions

Macrophages play a crucial role in the pathogenesis and resolution of IA diseases through their pro-inflammatory M1 and anti-inflammatory M2 phenotypes. The RA synovial microenvironment has a predominance of M1 macrophages, whereas PsA has higher levels of M2 macrophages in the synovium. Macrophage polarisation is a complex process and exists as a continuum with diverse intermediate phenotypes involving pro-inflammatory and anti-inflammatory mediators. Synovial macrophages often share characteristics of M1 and M2 phenotypes by expressing both markers in different proportions, thereby highlighting their plasticity. Macrophage plasticity is a central characteristic of these immune cells’ function and phenotype with the potential existence of more subtypes and ability to repolarise into different subtypes. Since there are currently no therapeutic treatments targeting macrophages specifically in clinics, novel treatments are a priority. Targeting the imbalance in the M1/M2 subtypes ratio whilst sparing other subsets and re-establishing homeostasis is necessary for inflammation resolution, regeneration and the treatment of IA.

## Figures and Tables

**Table 1:** Table highlighting the differences between macrophages in RA and PsA. Patients with PsA demonstrate similar levels of M2 macrophage expression in comparison to RA; however, they have a lower expression of M1 inflammatory cytokines [30]. The M2 macrophages in PsA are said to be dysfunctional due to their lower efficiency in suppressing DC activity and producing higher levels of IL-6 upon stimulation. Similar signalling pathways are involved in RA and PsA; however, IL-17A involvement in PsA indicates a unique inflammatory signature.

	RA	PsA
Disease Characteristics	Progressive bone and joint destruction [12]	New bone formation via osteoclastogenesis after initial destructive processes [12]
Phenotype in Synovial Microenvironment	Predominantly pro-inflammatory M1 [3]	Dysfunctional M2 and pro-inflammatory M1 [30,31]
Macrophage Activation	Pathways involving TLR expression (TLR2, TL4) [32,33]	Pathways involving CD206 [34]
Signalling Pathways	NF-κB, JAK/STAT and MAPK [35,36]	NF-κB, JAK/STAT, MAPK and IL-17 [31]
Cytokine Expression	High IFNγ, TNF-α and IL-1β [30]	High CD163, VEGF, IL-6 and IL-17A [3,30,31]
Presence of Autoantibody	RF and ACPA [8]	No autoantibodies [37]
